# Total Flavonoids of *Scutellaria barbata* Inhibit Invasion of Hepatocarcinoma via MMP/TIMP *in Vitro*

**DOI:** 10.3390/molecules18010934

**Published:** 2013-01-11

**Authors:** Zhi-Jun Dai, Bao-Feng Wang, Wang-Feng Lu, Zhi-Dong Wang, Xiao-Bin Ma, Wei-Li Min, Hua-Feng Kang, Xi-Jing Wang, Wen-Ying Wu

**Affiliations:** 1 Department of Oncology, the Second Affiliated Hospital, Medical School of Xi’an Jiaotong University, Xi’an 710004, China; 2 Department of General Surgery, the Second Affiliated Hospital, Medical School of Xi’an Jiaotong University, Xi’an 710004, China; 3 Department of Pharmacology, the Second Affiliated Hospital, Medical School of Xi’an Jiaotong University, Xi’an 710004, China

**Keywords:** hepatoma, *Scutellaria barbata*, invasion, metastasis, matrix metalloproteinase

## Abstract

Metastasis is the major cause of cancer-related deaths. Targeting the process of metastasis has been proposed as a strategy to fight cancer. *Scutellaria barbata* D. Don (*S. barbata*), a traditional Chinese medicine, is used for treatment of many diseases, including cancer. This study aimed to determine the anti-metastatic effect of total flavonoids of *S. barbata* (TF-SB) using the human hepatocarcinoma MHCC97H cell line with high metastatic potential. Our results show that TF-SB could significantly inhibit the proliferation and invasion of MHCC97H cells in a dose-dependent manner. MMP-2 and MMP-9 expression were obviously decreased after TF-SB treatment at both the mRNA and protein level. TIMP-1 and TIMP-2 expression were simultaneously increased. The present study indicates that TF-SB could reduce the metastatic capability of MHCC97H cell, probably through decrease of the MMP expression, and simultaneous increase of the TIMP expression.

## 1. Introduction

Hepatocarcinoma is one of the most common cancers, with high incidence and mortality. Curative surgery or liver transplantation are rarely possible and often lead to tumor recurrence [1]. Although many therapy strategies for hepatocarcinoma exist, the therapeutic outcome remains very poor [2]. Tissue invasion and metastasis are the primary cause of the mortality of hepatocarcinoma patients. Therefore, to develop candidate drugs that target the process of metastasis is very important. Many Chinese herbs used in folklore medicine continue to be an important source of discovery and development of novel or potential therapeutic agents for the treatment of cancer [3,4].

Metastasis is a multi-step process, including local invasion, lymphatic and blood vascular system, survival in the bloodstream, extravascularizations from the microvessels and colonization at the secondary site [5,6]. This process depends on the activities of many factors associated with the proteolytic degradation of extracellular matrix (ECM) components [7]. Matrix metalloproteinases (MMPs) are a class of zinc-dependent endopeptidase enzymes, that play a crucial role in ECM degradation and tumor cell invasion, metastasis and angiogenesis [7,8]. MMP-2 and MMP-9 degrade most of the ECM components of basal membrane and type IV collagen, a major component of the basement membrane. Activities of MMPs are controlled by their endogenous inhibitors, metalloproteinases (TIMPs) such as TIMP-1 and TIMP-2, in cancer cells [9]. It was reported that when the balance of MMPs and TIMPs was broken, direct inhibition of MMPs and increase of TIMPs in cancer may be a particularly attractive target for therapeutic intervention in tumor invasion and metastasis [10].

*Scutellaria barbata* D. Don (*S. barbata*) is a perennial herb which mainly grows throughout southern China. The *S. barbata* herb is known in traditional Chinese medicine as Ban-Zhi-Lian, and has been used as an anti-inflammatory and antitumor agent as well as a diuretic in China and Korea [11,12,13,14,15,16,17,18,19]. *S. barbata* contains a large number of alkaloids, flavones, steroids, and polysaccharides [20,21,22,23]. The herb has been used in clinics in treating lung cancer, digestive system cancers, hepatoma, breast cancer, and chorioepithelioma. *S. barbata* D. Don (CE-SB) extracts have *in vitro* growth inhibitory effects on numerous human cancers including leukemia, colon cancer, hepatoma, and skin cancer [14,15,16,17,18]. Furthermore, our previous research found that CE-SB crude extracts have antitumor activities both *in vitro* and *in vivo* [24]. In the present study, we investigated the anti-metastatic effect of total flavonoids of *S. barbata* (TF-SB) using the human hepatocarcinoma MHCC97H cell line to clarify the possible molecular mechanism of TF-SB in inhibiting cancer cell invasion.

## 2. Results and Discussion

### 2.1. TF-SB Inhibits Proliferation in MHCC97H Cells

The ethanol extracts of *S. barbata* greatly inhibited A549 cell growth, with an IC_50_ of 0.21 mg/mL [15]. Bezielle, an aqueous extract of *S. barbata*, inhibited cell proliferation, induced cell death and G2 cycle arrest by regulating the mediator proteins Jab1, p27(Kip1) and p21(Cip1) in breast cancer cells [25]. It was reported by Marconett *et al*. that Bezielle exerts phenotype specific anti-proliferative gene expression responses in human breast and prostate cancer cells [26].

**Figure 1:**
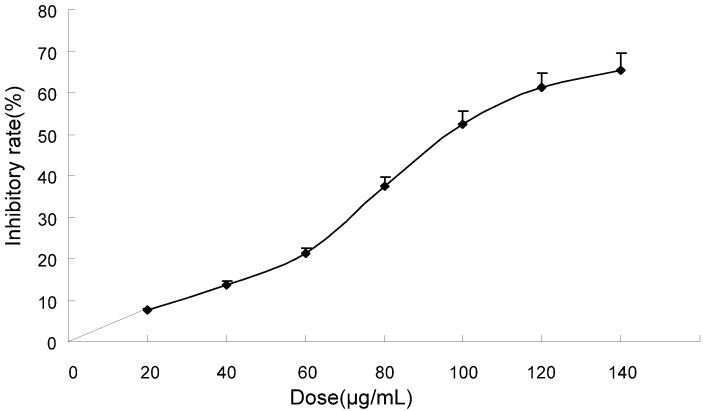
Growth inhibiting effects of TF-SB on MHCC97H cells. MHCC97H cells were treated with TF-SB (0, 20, 40, 60, 80, 100, 120 and 140 μg/mL) for 24 h. Cell viability was determined by MTT method. This assay was performed in triplicate. Dose-dependent inhibition of cell growth could be observed (*p* < 0.05, ANOVA analysis).

In this study, MHCC97H cells were treated with different doses of TF-SB. The 3-(4,5)-dimethylthiazol-y1-3,5-diphenyltetrazolium bromide (MTT) assay was used to examine the anti-proliferative effect of TF-SB on MHCC97H cells. The effects of 0–140 μg/mL TF-SB on cell growth after 24 h are shown in Figure 1. After 24 h of incubation, TF-SB significantly suppressed MHCC97H cell growth in a dose-dependent manner, with cell numbers markedly reduced compared to control. As shown in Figure 1, the inhibitory rate of TF-SB on cell growth was as 7.5, 13.8, 21.2, 37.4, 52.5, 61.2 and 65.5%, respectively.

### 2.2. TF-SB Inhibits Migration and Invasion of MHCC97H Cells

Metastasis of malignant tumors is a major cause of morbidity and mortality. Inhibition of tumor growth in distant organs is of clinical importance [6]. Cancer cell migration and invasion are two important steps in cancer metastasis [27]. The MHCC97H cell line is a typical human liver cancer cell with high metastatic potential [28,29], commonly used in the study of antitumor invasion agents.

*S. barbata* could inhibit the development of preneoplastic lesions in carcinogen-treated mouse mammary glands in culture and inhibited tumorigenesis in a mouse skin cancer model [17]. Bezielle inhibits breast cancer cell lines by inducing apoptosis *in vitro*. In a phase I clinical trial in the USA, Bezielle was safe and had a favorable toxicity profile [30]. The result of an open-label, phase IB, multicenter trial demonstrated that oral administration of Bezielle was safe, well tolerated, and showed promising clinical evidence of anticancer activity in this heavily pretreated population of women with metastatic breast cancer [31]. However, the anti-metastatic molecular mechanism of *S. barbata* was unclear.

**Figure 2:**
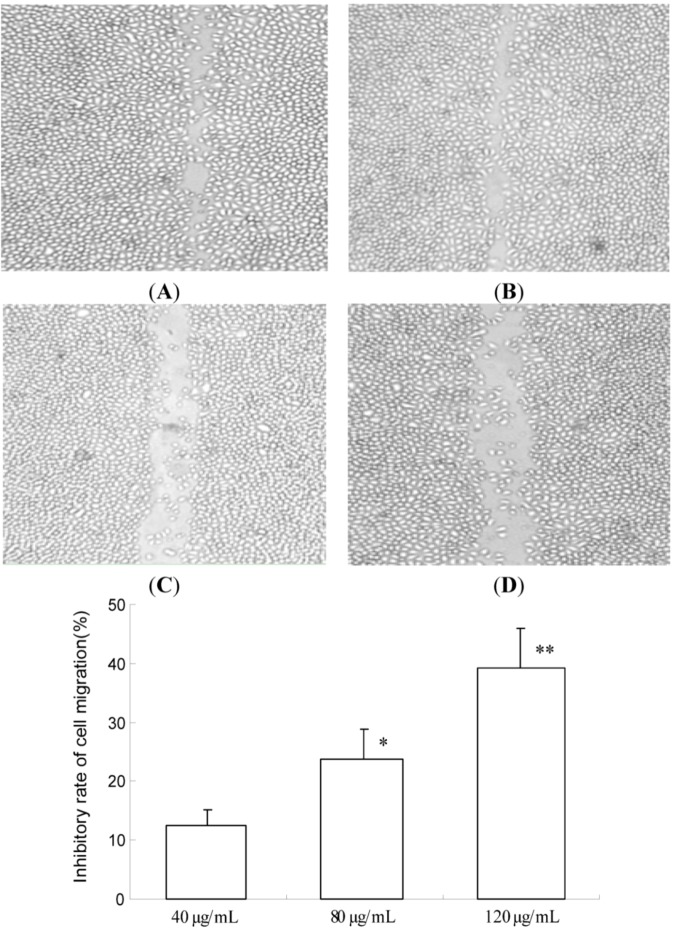
Effects of TF-SB on migration of MHCC97H cells. Cell migration was analyzed by wound healing assay. MHCC97H cells were treated with various concentrations of TF-SB (40, 80 and 120 μg/mL) for 24 h. Values represent mean ± SEM from three independent experiments. (**A**)blank control group; (**B**) 40 μg/mLTF-SB group; (**C**) 80 μg/mLTF-SB group; (**D**) 120 μg/mLTF-SB group. * *p* < 0.05, ** *p* < 0.01 versus 40 μg/mLTF-SB group.

In this study, an *in vitro* migration assay was used to investigate the inhibitory effect of TF-SB. The potential effect of TF-SB on cell migration was tested by counting MHCC97H cells that migrated through a matrigel coated-membrane. As shown in Figure 2, inhibitory rate of TF-SB (40, 80 and 120 μg/mL) on cell migration were 12.5 ± 2.6, 23.7 ± 5.1 and 39.2 ± 7.3, respectively. A significant decrease in the number of cancer cells migrating through the filters was observed after treatment with TF-SB (40, 80 and 120 μg/mL) for 24 h (*p* < 0.01).

**Figure 3:**
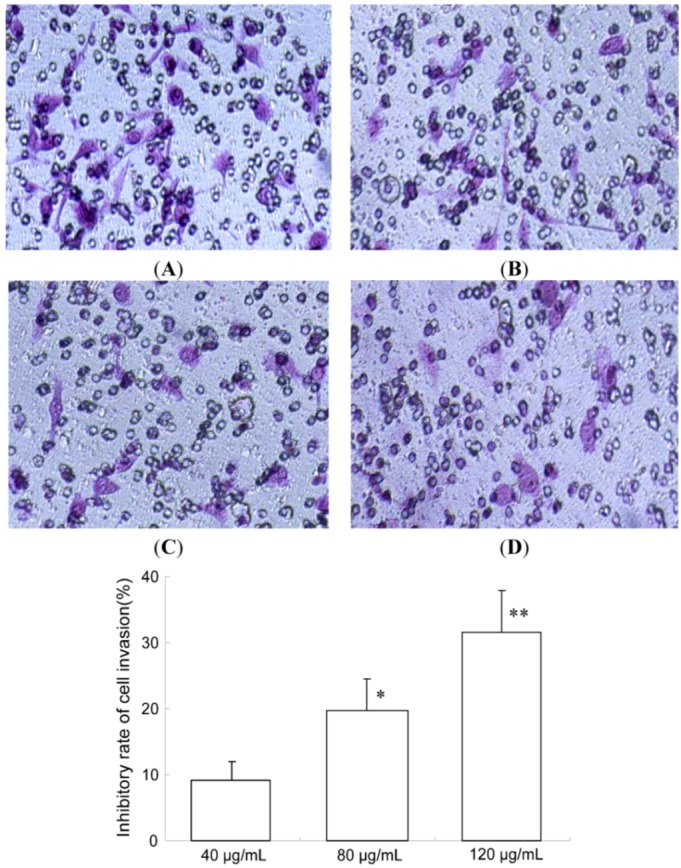
Effects of TF-SB on cell invasion of MHCC97H cells. Cell invasion was determined by transwell assay. MHCC97H cells were treated with various concentrations of TF-SB (40, 80 and 120 μg/mL) for 24 h. Cells in the lower chamber were then stained and quantified. For each plate, a representative number of invaded cells were counted under the microscope and averaged in five random fields at ×200 magnification. Bars represent mean ± SEM of these averages from triplicate plates. (**A**)blank control group; (**B**) 40 μg/mLTF-SB group; (**C**) 80 μg/mLTF-SB group; (**D**) 120 μg/mLTF-SB group. (* *p* < 0.05, ** *p* < 0.01 versus 40 μg/mLTF-SB group).

Furthermore, the effect of TF-SB on MHCC97H cells invasion was determined in a transwell chamber and basement membrane matrigel invasion assay. Quantitative data derived from three independent experiments shows that TF-SB effectively inhibited the cell invasion in this assay. As shown in Figure 3, inhibitory rate of TF-SB (40, 80 and 120 μg/mL) on cell invasion were 9.2 ± 2.8, 19.7 ± 4.8 and 31.6 ± 6.5, respectively. The results suggest that TF-SB inhibits migration and invasion of MHCC97H Cells in a dose -dependent manner.

### 2.3. TF-SB Reduces MMP-2 and MMP-9 mRNA Expression in MHCC97H Cells with RT-PCR Assay

The process of metastasis includes separation of tumor cells from the original niche, invasion of underlying basal lamina, entry to the cardiovascular or lymphatic circulation and formation of secondary lesions [5,6]. Degradation and remodeling of extracellular matrix (ECM) are necessary steps in local invasion. Excess ECM degradation is one of the hallmarks of tumor migration. MMP-2 (gelatinase-A) and MMP-9 (gelatinase-B) degrade most of the ECM components of basal membrane and type IV collagen. Increased expression and activity of MMP-2 has been well characterized in human HCC [4]. Basal membrane degradation correlated positively with MMP-2 and MMP-9 proteolytic activity [10]. Therefore, the inhibition of migration or invasion mediated by MMPs might represent a strategy for preventing hepatocarcinoma metastasis [5].

**Figure 4:**
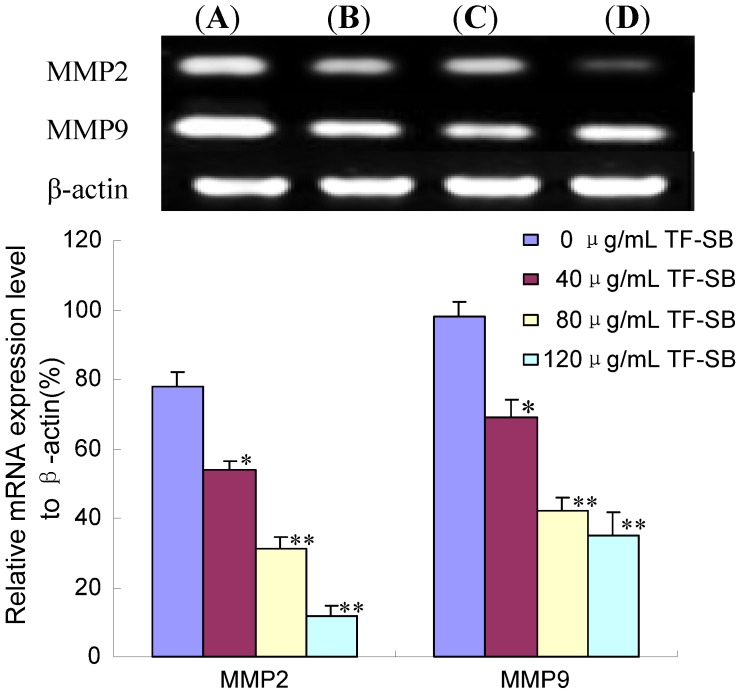
Effect of TF-SB on MMP-2 and MMP-9 mRNA expression in MHCC97H cells. MHCC97H cells were treated with various concentrations of TF-SB (40, 80 and 120 μg/mL) for 24 h, and then the mRNA expression of MMP-2 and MMP-9 was identified by RT-PCR assay. (**A**)blank control group; (**B**) 40 μg/mLTF-SB group; (**C**) 80 μg/mLTF-SB group; (**D**) 120 μg/mLTF-SB group. Values represent means ± SEM. This assay was done triplicate (* *p* < 0.05, ** *p* < 0.01 versus control group).

MMP-2 and MMP-9 are significantly up-regulated in malignant tumors and cause invasion and metastatic spread of cancer cells [32]. It was reported that MMPs highly expressed in MHCC97H cells [27,29]. In the present study, we tested the effect of TF-SB on the expression of MMP-2 and MMP-9 to investigate the possible molecular mechanisms of anti-metastasis. The cells were pretreated with concentrations of TF-SB (80 μg/mL) for 24 h, and then MMP-2 and MMP-9 expression was identified by RT-PCR assay. As shown in Figure 4, there was a significant increase in MMP-2 and MMP-9 mRNA expression in MHCC97H cells treated with 40, 80 and 120 μg/mL TF-SB in comparison with the control group (*p* < 0.05). Furthermore, mRNA expression of MMP-2 and MMP-9 in 120 μg/mL TF-SB group was decreased 550% and 180%. As shown in Figure 4, RT-PCR results showed that TF-SB markedly suppressed MMP-2 and MMP-9 protein expression in a dose-dependent manner (*p* < 0.05).

### 2.4. TF-SB Decreased MMP-2 and MMP-9 Protein Expression with Immunofluorescence and Western Blot Analysis

**Figure 5:**
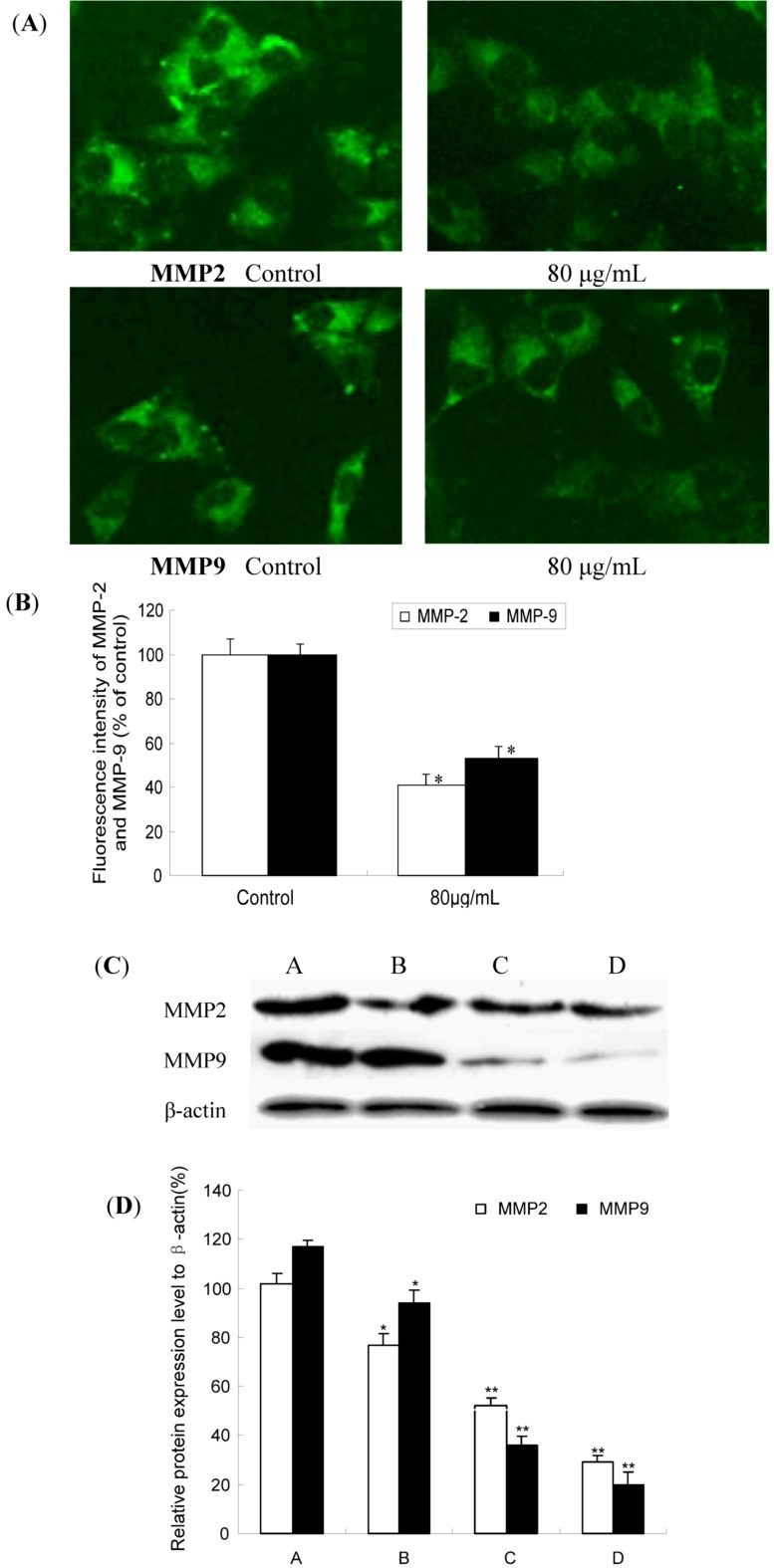
Effect of TF-SB on MMP-2 and MMP-9 protein expression in MHCC97H cells. MHCC97H cells were treated with various concentrations of TF-SB (40, 80 and 120 μg/mL) for 24 h. The protein expression of MMP-2 and MMP-9 were determined by immunofluorescence and Western blot analysis. (**A**) blank control group; (**B**) 40 μg/mL TF-SB group; (**C**) 80 μg/mL TF-SB group; (**D**) 120 μg/mL TF-SB group. Values represent means ± SEM. This assay was done triplicate (* *p* < 0.05, ** *p* < 0.01 versus blank control group).

Prompted from the above results by RT-PCR method, we further analyzed the effect of TF-SB on MMP-2 and MMP-9 protein expression in MHCC97H cells. When MHCC97H cells were treated with TF-SB (0, 40, 80 and 120 μg/mL) for 24 h, the amount of MMP-2 and MMP-9 protein expression were measured by immunofluorescence and Western blot analysis. In immunofluorescence analysis, MHCC97H cells were treated with 80 μg/mL TF-SB for 24 h, and then the expression of MMP-2 and MMP-9 was identified by immunocytofluorescence assay and observed under a fluorescence microscope. Fluorescence intensity of MMP-2 and MMP-9 was also detected. As shown in Figure 5A,B, compared with control group (100%), MMP-2 and MMP-9 expression of 80 μg/mL TF-SB group were suppressed by 57% and 44%, respectively(*p* < 0.01). As shown in Figure 5C,D, Western blot results showed that TF-SB obviously inhibited MMP-2 and MMP-9 protein expression in a dose-dependent manner (*p* < 0.05).

### 2.5. TF-SB Promotes TIMP-1 and TIMP-2 mRNA Expression in MHCC97H Cells with RT-PCR Assay

Activities of MMPs are controlled by their endogenous inhibitors, metalloproteinases (TIMPs) such as TIMP-1 and TIMP-2 in cancer cells [9]. Decreased expression of MMP-2 in tumors was paralleled by the elevated levels of metalloproteinase inhibitor TIMP-1 and TIMP-2 [33].

**Figure 6:**
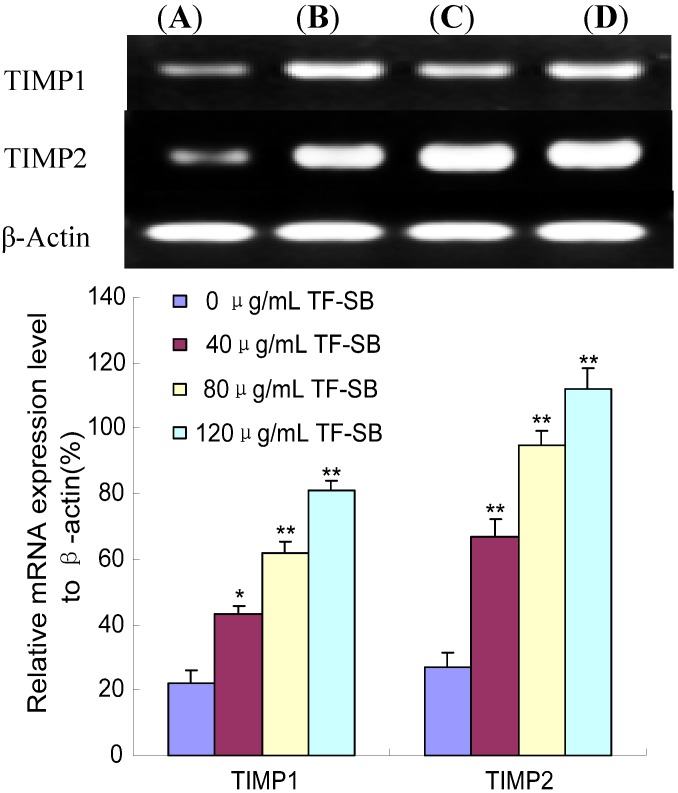
Effect of TF-SB on TIMP-1 and TIMP-2 mRNA expression in MHCC97H cells. MHCC97H cells were treated with various concentrations of TF-SB (40, 80 and 120 μg/mL) for 24 h, and then the mRNA expression of MMP-2 and MMP-9 was identified by RT-PCR assay. (**A**) blank control group; (**B**) 40 μg/mLTF-SB group; (**C**) 80 μg/mLTF-SB group; (**D**) 120 μg/mLTF-SB group. Values represent means ± SEM. This assay was done triplicate (* *p* < 0.05, ** *p* < 0.01 versus blank control group).

As mentioned above, the MMP-2 and MMP-9 expression was decreased after TF-SB treatment. To confirm whether TF-SB inhibits MMPs production via increasing TIMP expression, expression of TIMP-2 and TIMP-9 in MHCC97H cells were identified by RT-PCR assay. As shown in Figure 6, there was a significant increase in TIMP-1 and TIMP-2 mRNA expression in MHCC97H cells treated with 40, 80 and 120 μg/mL TF-SB in comparison with the control group (*p* < 0.05). Furthermore, TIMP-1 and TIMP-2 mRNA expression of 120 μg/mL TF-SB group were enhanced 258% and 315%.

### 2.6. TF-SB Increases TIMP-1 and TIMP-2 Protein Expression with Immunofluorescence and Western Blot Analysis

**Figure 7:**
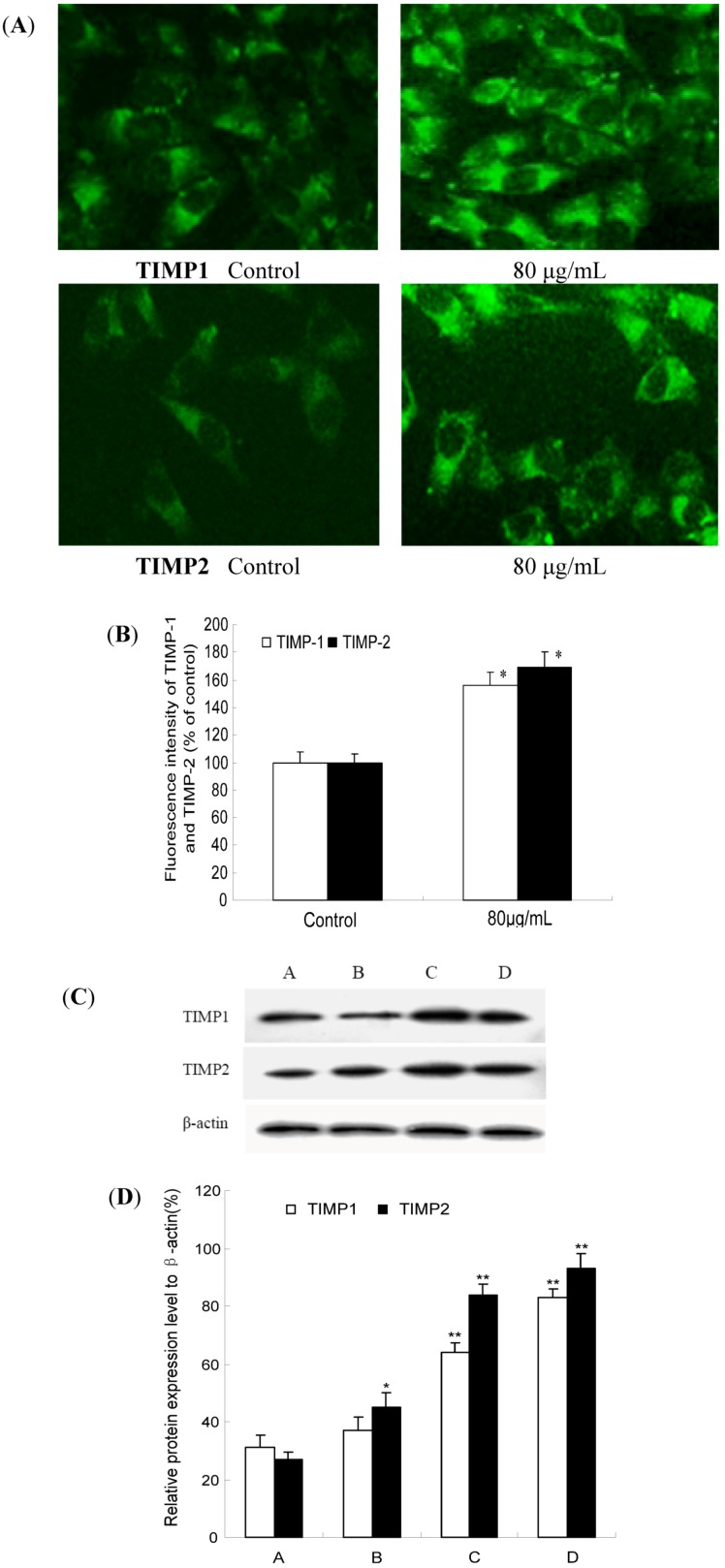
Effect of TF-SB on TIMP-1 and TIMP-2 protein expression in MHCC97H cells. MHCC97H cells were treated with various concentrations of TF-SB (40, 80 and 120 μg/mL) for 24 h. The protein expression of TIMP-1 and TIMP-2 were determined by immunofluorescence and Western blot analysis. (**A**) blank control group; (**B**) 40 μg/mLTF-SB group; (**C**) 80 μg/mLTF-SB group; (**D**) 120 μg/mLTF-SB group. Values represent means ± SEM. This assay was done triplicate (* *p* < 0.05, ** *p* < 0.01 versus blank control group).

TIMPs are preferably bound to the active center and inhibit MMP protease activity, thereby suppressing tumor invasion and metastasis. The balance between both molecules finally determines the net proteolytic activity. To examine whether TF-SB had decreased MMPs and increased simultaneously the expression of their natural inhibitors or not, we tested TIMP-1 and TIMP-2 protein expression with immunofluorescence and Western blot analysis. In immunofluorescence analysis, MHCC97H cells were treated with 80 μg/mL TF-SB for 24 h, and then the expression of MMP-2 and MMP-9 was identified by immunocytofluorescence assay and observed under a fluorescence microscope. As shown in Figure 7(A,B), compared with the control group (100%), TIMP-1 and TIMP-2 expression of 80 μg/mL TF-SB were increased by 59% and 66%, respectively (*p* < 0.01).

As shown in Figure 7(C,D), Western blot results showed that both TIMP-1 and TIMP-2 protein expression significantly increased in TF-SB treated group comparing with the control group (*p* < 0.05). Furthermore, this enhancement effect was in a dose-dependent manner (*p* < 0.05).

## 3. Experimental

### 3.1. Reagents

Fetal bovine serum (Gibco BRL, Rockville, MD, USA); RPMI1640 medium (Gibco); 3-(4,5)-dimethylthiazoly1-3,5-diphenytetrazolium bromide (MTT) (Gibco); Transwell (Corning Costar, Cambridge, MA, USA); Matrigel (BD Biosciences, San Jose, CA, USA); RT-PCR kit (Ampliqon, Herlev, Denmark); Trizol (Invitrogen, Carlsbad, CA, USA); Anti-MMP-2, Anti-MMP-9, Anti-TIMP-1, Anti-TIMP-2 and anti-β-actin were purchased from Santa Cruz Biotechnology (Santa Cruz, CA, USA).

### 3.2. Cell Line and Cell Culture

Human MHCC97H, a hepatocellular carcinoma cell line with high metastatic potential, was obtained from Liver Cancer Institute of Fudan University (Shanghai, China). Cells were cultured in RPMI 1640 maximal medium containing 10% inactived fetal bovine serum in a humidified atmosphere with 5% CO_2_ incubator at 37 °C.

### 3.3. Preparation of TF-SB from Scutellaria barbata D. Don

Dried plant materials of *S. barbata* were purchased from Yi Shan Tang Chinese Herbal medicine store (Xi’an, China). The original herb was identified as *Scutellaria barbata* D. Don (SB) by Run-Xia Liu at Medical School of Xi’an Jiaotong University, Xi’an, China. The material was coarsely ground before extraction. A total of 300 g of the material was extracted two times with 95% ethanol for 3 h in 50 °C. The fluid was filtered through a 1-mm pore-size filter. Then the filtrate was evaporated. The crude extract were isolated by AB-8 macroporous adsorption resin column in which 70% aqueous ethanol was used to elute flavonoids.

### 3.4. MTT Assay for the Proliferation of MHCC97H Cells

Viability of MHCC97H cells was assessed using the MTT dye reduction assay (Sigma, St. louis, Mo, USA), which was conducted as described previously [34]. Cells were seeded in a 96-well plate at a density of 1 × 10^4^ cells/well, cultured for 12 h, then treated with different concentration (0, 40, 80 and 120 μg/mL) TF-SB for 0–96 h. At the end of the treatment, MTT, 50 μg/10 μL, was added and the cells were incubated for another 4 h. Dimethyl sulfoxide (DMSO; 200 μL) was added to each well after removal of the supernatant. After shaking the plate for 10 min, cell viability was assessed by measuring the absorbance at 490 nm using an enzyme-labeling instrument (ELX800, Bio-Tek, Winooski, VT , USA); all measurements were performed three times. The results represented as the average of three independent experiments done over multiple days.

The growth inhibition was calculated according to the following formula: The Growth Inhibition Ratio (IR%) = [(the absorbance of blank control group − the absorbance of experimental group)/the absorbance of blank control group] × 100%.

### 3.5. Wound Healing Assay

Cell migration assay was performed as previously described [35]. In brief, MHCC97H cells were grown to confluence in a 6-well culture plate to a density of approximately 5 × 10^6^ cells/well. A wound was created by scratching cells with a sterile 200 μL pipette tip. The plates were washed twice with PBS and then replaced with complete RPMI-1640 medium. After treated with different concentration of TF-SB (0, 40, 80 and 120 μg/mL) for 24 h, the cells were denuded by dragging a rubber policeman (Fisher STF-Scientific, Hampton, NH, USA) through the center of the plate. Photographs were taken at 0 and 24 h, respectively. The level of cell migration was analyzed with NIH Image software (Image J). The cells migration rate was obtained by counting three fields per area and represented as the average of three independent experiments done over multiple days.

The inhibition rate of cell migration was calculated according to the following formula: Inhibition Ratio (IR%) = [(the number of cells migrating through the filters in blank control group − the number of cells migrating through the filters in experimental group)/the number of cells migrating through the filters in blank control group] × 100%.

### 3.6. Invasion Assay

We used Boyden chamber invasion assay to measure the ability of MHCC97H cells to pass through filters coated with matrigel [33]. Briefly, matrigel basement membrane matrix was diluted to 200 μg/mL in cold serum-free medium and used to coat the upper side of 8 μm pore transwell filters (BD Biosciences). Before the invasion assay, MHCC97H cells were treated with various concentrations of TF-SB (0, 40, 80 and 120 μg/mL) for 24 h, detached from the culture plates and resuspended in serum-free RPMI-1640 medium (1 × 10^5^ cells/200 μL). The cells were then seeded in the upper chamber with a serum-containing medium (500 μL) simultaneously added to the lower chamber for 24 h. After incubation, the cells on the upper surface of the filter were removed with cotton swabs. Cells invading across the matrigel to the lower surface of the membrane were stained withand Cells that passed through the matrigel-coated membrane were stained with 0.1% crystal violet (in 20% ethanol) and photographed. The values for invasion were obtained by counting three fields per membrane with light microscope under 200× magnification. Each experiment was carried out in triplicate. The inhibition rate of cell invasion was calculated according to the following formula: Inhibition Ratio (IR%) = [(the number of cells migrating through the filters in blank control group—the number of cells migrating through the filters in experimental group)/the number of cells migrating through the filters in blank control group] × 100%.

### 3.7. Reverse Transcription-Polymerase Chain Reaction (RT-PCR)

MHCC97H cells were seeded in 6 cm culture capsules and treated with various concentration of TF-SB (0, 40, 80 and 120 μg/mL) separately for 24 h. Each group contained three culture capsules. As previously described [36], cells collected at specified time were used to extract total RNA using the Trizol reagent following the manufacturer’s instructions. 1 μg RNA synthetized cDNA through reverse transcriptase underwent the following treatment conditions: 70 °C 5 min, 42 °C extended for 60 min, 95 °C enzyme inactivated for 3 min and 4 °C terminated reaction. Synthetic cDNA as template to carry out polymerase chain reaction. MMP2 primer sequence (Invitrogen): 5'-GACGGTAAGGACGGACTC-3' (sense) and 5'-TGCCATTGAACAAGAAGGG-3' (anti-sense), amplification fragment was 172 bp, renaturation temperature was 55 °C (cycling 25 times). MMP9 primer sequence (Invitrogen): 5'-AGAGATGCGTGGAGAGTC-3' (sense) and 5'-GGTGATGTTGTGGTGGTG-3' (anti-sense), amplification fragment was 193 bp. TIMP1 primer sequence (Invitrogen): 5'-CCTGTTGTTGC TGTGGCTGA-3' (sense) and 5'-CATAACGCTGGTATAAGGTGGTCTG-3' (anti-sense), amplification fragment was 152 bp. Beclin 1 primer sequence (Invitrogen): 5'-AGCAGATAAAGA TGTTCAAAGG-3' (sense) and 5'-CACGATGAAGTCACAGAGG-3' (anti-sense), amplification fragment was 173 bp. β-actin primer sequence was 5'-ATCGTGCGTG ACATTAAGGAGAAG-3' (sense), 5'-AGGAAGGAAGGCTGGAAGAGTG-3' (anti-sense), amplification fragment was 179 bp. Renaturation temperature was 55 °C (cycling 20–25 times). Amplification condition was below: pre-denaturized for 3 min at 95 °C, denaturized for 30 s at 95 °C, renaturated for 30 s at 55 °C and extended for 30 s at 72 °C. PCR product was detected on agarose gel electrophoresis and ethidium bromide imaging system was used to make density index analysis. The expression intensity of destination gene mRNA was denoted with the ratio of the photodensity of the RT-PCR products of destination gene and β-actin.

### 3.8. Immunofluorescence Staining

To detect the effect of TF-SB on MMP-2, MMP-9, TIMP-1 and TIMP-2 expression in MHCC97H cells, the cells were pretreated with TF-SB (0, 80 μg/mL) for 24 h. After the treatment, the cells were fixed with 4% paraformaldehyde followed by 0.2% Triton X-100, PBS washing, and nonspecific binding sites were blocked with 10% goat serum at 37 °C for 30 min. The cells were incubated with anti-MMP-2, anti-MMP-9, anti-TIMP-1 and anti-TIMP-2(1:500; Santa Cruz Biotechnology) at 4 °C overnight and then incubated with the appropriate fluorescence-labeled secondary antibody conjugated to fluorescein isothiocyanate (FITC) at room. The expression of target genes in MHCC97H cells were observed under a fluorescence microscope (Olympus, BX-60, Tokyo, Japan).

### 3.9. Western Blot Analysis

MHCC97H cells were treated with various concentration of TF-SB (0, 40, 80 and 120 μg/mL) for an appropriate time. As previously described [34], cells were lysed in a sample buffer followed by denaturation. Protein concentrations were determined using the PIERCE BCA protein assay kit and equal amounts of protein (50 μg) were subjected to SDS-PAGE on 10% gel. The proteins were then electrophoretically transferred to nitrocellulose membranes. The membranes were blocked with 5% skimmed milk, respectively incubated with anti-MMP-2, anti-MMP-9, anti-TIMP-1 and anti-TIMP-2 (1:500; Santa Cruz Biotechnology) at 4 °C overnight. And then followed by incubation in goat antimouse secondary antibody conjugated with horseradish peroxidase (1:1000; Santa Cruz Biotechnology). Equal loading of each lane was evaluated by immunoblotting the same membranes with β-actin antibodies after detachment of previous primary antibodies. Photographs were taken and optical densities of the bands were scanned and quantified with the Gel Doc 2000 (Bio-Rad).

### 3.10. Statistical Analysis

Data were expressed as mean ± SEM. Statistical analysis was performed with Student *t* tests and one-way analysis of variance (ANOVA) test using the statistical software SPSS 13.0. *p* values less than 0.05 were considered statistically significant.

## 4. Conclusions

In conclusion, TF-SB could significantly inhibit the proliferation and invasion of MHCC97H cells in a dose-dependent manner. Importantly, our data suggests that the effect of TF-SB on tumor growth and invasion might occur through decreasing expression of MMPs, and increasing expression of TIMP. In the future, additional experiments will be required to determine the exact mechanism involved in the antimetastatic effects of TF-SB.

## References

[1] Jemal A., Bray F., Center M.M., Ferlay J., Ward E., Forman D. (2011). Global cancer statistics. CA Cancer J. Clin..

[2] Farazi1 P.A., DePinho R.A. (2006). Hepatocellular carcinoma pathogenesis: From genes to environment. Nat. Rev. Cancer.

[3] Vickers A. (2002). Botanical medicines for the treatment of cancer: Rationale, overview of current data, and methodological considerations for phase I and II trials. Cancer Invest..

[4] Wang Z.D., Huang C., Li Z.F., Yang J., Li B.H., Liang R.R., Dai Z.J., Liu Z.W. (2010). Chrysanthemum indicum ethanolic extract inhibits invasion of hepatocellular carcinoma via regulation of MMP/TIMP balance as therapeutic target. Oncol. Rep..

[5] Bjorklund M., Koivunen E. (1755). Gelatinase-mediated migration and invasion of cancer cells. Biochim. Biophys. Acta.

[6] Khan S.T., Pixley R.A., Liu Y., Bakdash N., Gordon B., Agelan A., Huang Y., Achary M.P., Colman R.W. (2010). Inhibition of metastasis of syngeneic murine melanoma *in vivo* and vasculogenesis *in vitro* by monoclonal antibody C11C1 targeted to domain 5 of high molecular weight kininogen. Cancer Immunol. Immunother..

[7] Kang J.H., Han I.H., Sung M.K., Yoo H., Kim Y.K., Kim J.S., Kawada T., Yu R. (2008). Soybean saponin inhibits tumor cell metastasis by modulating expressions of MMP-2, MMP-9 and TIMP-2. Cancer Lett..

[8] Hu Y.H., Yu L.J., Shao E.D., Wu J.L., Ji J.W. (2009). The regulating role of mutant IkappaBalpha in expression of TIMP-2 and MMP-9 in human glioblastoma multiform. Chin. Med. J. (Engl.).

[9] Figueira R.C., Gomes L.R., Neto J.S., Silva F.C., Silva I.D., Sogayar M.C. (2009). Correlation between MMPs and their inhibitors in breast cancer tumor tissue specimens and in cell lines with different metastatic potential. BMC. Cancer.

[10] Giannelli G., Bergamini C., Marinosci F., Fransvea E., Quaranta M., Lupo L., Schiraldi O., Antonaci S. (2002). Clinical role of MMP-2/TIMP-2 imbalance in hepatocellular carcinoma. Int. J. Cancer.

[11] Lee T.K., Lee D.K., Kim D.I., Lee Y.C., Chang Y.C., Kim C.H. (2004). Inhibitory effects of *Scutellaria barbata* D. Don on human uterine leiomyomal smooth muscle cell proliferation through cell cycle analysis. Int. Immunopharmacol..

[12] Lin C.C., Shieh D.E. (1996). The anti-inflammatory activity of *Scutellaria rivularis* extracts and its active components, baicalin, baicaleinand wogonin. Am. J. Chin. Med..

[13] Lee T.K., Kim D.I., Song Y.L., Lee Y.C., Kim H.M., Kim C.H. (2004). Differential inhibition of *Scutellaria barbata* D. Don (Lamiaceae) on HCG-promoted proliferation of cultured uterine leiomyomal and myometrial smooth muscle cells. Immunopharmacol. Immunotoxicol..

[14] Goh D., Lee Y.H., Ong E.S. (2005). Inhibitory effects of a chemically standardized extract from *Scutellaria barbata* in human colon cancer cell lines, LoVo. J. Agric. Food Chem..

[15] Yin X., Zhou J., Jie C., Xing D., Zhang Y. (2004). Anticanceractivity and mechanism of Scutellaria barbata extract on human lung cancer cell line A549. Life Sci..

[16] Cha Y.Y., Lee E.O., Lee H.J., Park Y.D., Ko S.G., Kim D.H., Kim H.M., Kang I.C., Kim S.H. (2004). Methylene chloride fraction of *Scutellaria barbata* induces apoptosis in human U937 leukemia cells via the mitochondrial signaling pathway. Clin. Chim. Acta.

[17] Suh S.J., Yoon J.W., Lee T.K., Jin U.H., Kim S.L., Kim M.S., Kwon D.Y., Lee Y.C., Kim C.H. (2007). Chemoprevention of *Scutellaria bardata* on Human Cancer Cells and Tumorigenesis in Skin Cancer. Phytother Res..

[18] Dai Z.J., Wang X.J., Li Z.F., Ji Z.Z., Ren H.T., Tang W., Liu X.X., Kang H.F., Guan H.T., Song L.Q. (2008). *Scutellaria barbate* extract induces apoptosis of hepatoma H22 cells via the mitochondrial pathway involving caspase-3. World J. Gastroenterol..

[19] Lee T.K., Cho H.L., Kim D.I., Lee Y.C., Kim C.H. (2004). *Scutellaria barbata* D. Don induces c-fos gene expression in human uterine leiomyomal cells by activating beta2-adrenergic receptors. Int. J. Gynecol. Cancer.

[20] Dai S.J., Sun J.Y., Ren Y., Liu K., Shen L. (2007). Bioactive ent-clerodane diterpenoids from *Scutellaria barbata*. Planta Med..

[21] Qu G.W., Yue X.D., Li G.S., Yu Q.Y., Dai S.J. (2010). Two new cytotoxic ent-clerodane diterpenoids from *Scutellaria barbata*. J. Asian Nat. Prod. Res..

[22] Dai S.J., Peng W.B., Shen L., Zhang D.W., Ren Y. (2011). New norditerpenoid alkaloids from *Scutellaria barbata* with cytotoxic activities. Nat. Prod. Res..

[23] Mi X., Zhu R. (2010). Simultaneous determination of 7 active ingredients in *Scutellaria barbata* D. Don by capillary micellar electrokinetic chromatography. Se Pu.

[24] Dai Z.J., Gao J., Li Z.F., Ji Z.Z., Kang H.F., Guan H.T., Diao Y., Wang B.F., Wang X.J. (2011). *In vitro* and *in vivo* antitumor activity of *Scutellaria barbate* extract on murine liver cancer. Molecules.

[25] Klawitter J., Klawitter J., Gurshtein J., Corby K., Fong S., Tagliaferri M., Quattrochi L., Cohen I., Shtivelman E., Christians U. (2011). Bezielle (BZL101)-induced oxidative stress damage followed by redistribution of metabolic fluxes in breast cancer cells: A combined proteomic and metabolomic study. Int. J. Cancer.

[26] Marconett C.N., Morgenstern T.J., San-Roman A.K., Sundar S.N., Singhal A.K., Firestone G.L. (2010). BZL101, a phytochemical extract from the Scutellaria barbata plant, disrupts proliferation of human breast and prostate cancer cells through distinct mechanisms dependent on the cancer cell phenotype. Cancer Biol. Ther..

[27] Ji X.N., Ye S.L., Li Y., Tian B., Chen J., Gao D.M., Chen J., Bao W.H., Liu Y.K., Tang Z.Y. (2003). Contributions of lung tissue extracts to invasion and migration of human hepatocellular carcinoma cells with various metastatic potentials. J. Cancer Res. Clin. Oncol..

[28] Wang Z., Zhou J., Fan J., Tan C.J., Qiu S.J., Huang X.W., Tang Z.Y. (2009). Sirolimus inhibits the growth and metastatic progression of hepatocellular carcinoma. J. Cancer Res. Clin. Oncol..

[29] Huang X.Y., Wang L., Huang Z.L., Zheng Q., Li Q.S., Tang Z.Y. (2009). Herbal extract “Songyou Yin” inhibits tumor growth and prolongs survival in nude mice bearing human hepatocellular carcinoma xenograft with high metastatic potential. J. Cancer Res. Clin. Oncol..

[30] Rugo H., Shtivelman E., Perez A., Vogel C., Franco S., Tan C.E., Melisko M., Tagliaferri M., Cohen I., Shoemaker M. (2007). Phase I trial and antitumor effects of BZL101 for patients with advanced breast cancer. Breast Cancer Res. Treat..

[31] Perez A.T., Arun B., Tripathy D., Tagliaferri M.A., Shaw H.S., Kimmick G.G., Cohen I., Shtivelman E., Caygill K.A., Grady D. (2010). A phase IB dose escalation trial of Scutellaria barbata (BZL101) for patients with metastatic breast cancer. Breast Cancer Res. Treat..

[32] Kim S., Choi J.H., Kim J.B., Nam S.J., Yang J.H., Kim J.H., Lee J.E. (2008). Berberine suppresses TNF-α-induced MMP-9 and cell invasion through inhibition of AP-1 activity in MDA-MB-231 human breast cancer cells. Molecules.

[33] Han X., Yan D.M., Zhao X.F., Hiroshi M., Ding W.G., Li P., Jiang S., Du B.R., Du P.G., Zhu X. (2012). GHGKHKNK octapeptide (P-5m) inhibits metastasis of HCCLM3 cell lines via regulation of MMP-2 expression in *in vitro* and *in vivo* studies. Molecules.

[34] Dai Z.J., Gao J., Ma X.B., Yan K., Liu X.X., Kang H.F., Ji Z.Z., Guan H.T., Wang X.J. (2012). Up-regulation of hypoxia inducible factor-1α by cobalt chloride correlates with proliferation and apoptosis in PC-2 cells. J. Exp. Clin. Cancer Res..

[35] Lee K.J., Kim J.Y., Choi J.H., Kim H.G., Chung Y.C., Roh S.H., Jeong H.G. (2006). Inhibition of tumor invasion and metastasis by aqueous extract of the radix of *Platycodon grandiflorum*. Food Chem. Toxicol..

[36] Guan H.T., Xue X.H., Dai Z.J., Wang X.J., Li A., Qin Z.Y. (2006). Downregulation of survivin expression by small interfering RNA induces pancreatic cancer cell apoptosis and enhances its radiosensitivity. World J. Gastroenterol..

